# A System of Dental Surgery

**Published:** 1874-01

**Authors:** D. C. Hawxhurst


*A System of Dental Surgery:* By JOHN TOMES, F. R. S., Corresponding Member of the Philadelphia Academy of Natural Sciences, late Dental Surgeon to the Middlesex and Dental Hospitals; and CHAS. S. TOMES, M. A, Lecturer on Dental Anatomy and Physiology, and Assistant Dental Surgeon to the Hospital of London. Second Edition, Revised and Enlarged, with 863 Illustrations. Philadelphia: Lindsay and Blakiston, 1873.

This a manual of 734 closely printed pages. The type having been reduced in size since the last edition, the volume is not larger than the preceding, though much new matter has been added. Those parts which treat of neuralgia, dentigerous cysts, odontomes, along with other shorter portions scattered through the volume, are new. Sixty new illustrations have also made their appearance in this edition, which, when we consider the excellence of the plates, gives an unusually well-illustrated work.

It will seem a little odd to the American student to find the running title on the ffrst 239 pages to be "Teething." The word teething, however, is used by the author to cover the development of the teeth from the commencement to the completion of formative action. Accordingly, an extensive range of topics is treated under this head. The first 40 pages are devoted to the serial and progressive changes of form between the various parts of the jaws during infancy, and to their relations to the developing teeth of both dentitions. The conclusions reached are drawn from a graded collection of young skulls denuded of their soft tissues, which had been made by Chas. Tomes in the absence of any similar collection ; as we are told in the preface. The usefulness of the matter which is presented in this part of the book must be very great. The reactions of the first and second sets of teeth upon each other are so constant and varied in character, and the understanding of these influences is so necessary to him who is called upon to take charge of the eruption of children's teeth, that we feel like saying that this part of the work alone is worth the price of the volume. The next 200 pages are devoted to a great variety of topics : such as "Dentition, a Cause of Local and Constitutional Disturbance ;" "Relation of the Temporary to the Developing Permanent Teeth ;" "Causes of Irregularity ;" "Syphilitic Marks on the Teeth." It is all of much interest.

About 40 pages are now given to an exposition of the structure of the dental tissues, in which the minute structure of the teeth is well displayed. The remainder of the volume is more immediately adapted to the wants of the operator, containing an account of those conditions of the mouth which demand operative interference, with some account also of the methods employed by the distinguished authors in their own practice.

The later portions of this work contain many pathological hints of great value, with glimpses at the *rationale* of obscure pathological processes, that are original. The purely operative part will probably fail to meet the expectations of those who had looked for a description of better operative procedures than can be found in American works. Indeed, it would have been difficult, quite, to have excelled the descriptions of methods of operating which Prof. Taft has given us in his "Operative Dentistry." The latter work is direct in its statements, concise in style, thorough in the treatment of its subjects, and must long retain its place as a text-book for the student and companion to the operator. But, after having said this, it must still remain true that Tomes' manual will be sought by many of our best practitioners, for its admirable insight into physiology and pathology.

D. C. HAWXHURST.

